# MicroRNA: trends in clinical trials of cancer diagnosis and therapy strategies

**DOI:** 10.1038/s12276-023-01050-9

**Published:** 2023-07-10

**Authors:** Taewan Kim, Carlo M. Croce

**Affiliations:** 1 Department of Anatomy, Histology & Developmental Biology, International Cancer Center, School of Medicine, Shenzhen University, Shenzhen, China; 2 Department of Cancer Biology and Genetics, The Ohio State University, Columbus, OH USA

**Keywords:** Drug development, Cancer therapy

## Abstract

As a type of short noncoding RNAs, microRNA (miRNA) undoubtedly plays a crucial role in cancer development. Since the discovery of the identity and clinical functions of miRNAs, over the past few decades, the roles of miRNAs in cancer have been actively investigated. Numerous pieces of evidence indicate that miRNAs are pivotal factors in most types of cancer. Recent cancer research focused on miRNAs has identified and characterized a large cohort of miRNAs commonly dysregulated in cancer or exclusively dysregulated in specific types of cancer. These studies have suggested the potential of miRNAs as biomarkers in the diagnosis and prognostication of cancer. Moreover, many of these miRNAs have oncogenic or tumor-suppressive functions. MiRNAs have been the focus of research given their potential clinical applications as therapeutic targets. Currently, various oncology clinical trials using miRNAs in screening, diagnosis, and drug testing are underway. Although clinical trials studying miRNAs in various diseases have been reviewed before, there have been fewer clinical trials related to miRNAs in cancer. Furthermore, updated results of recent preclinical studies and clinical trials of miRNA biomarkers and drugs in cancer are needed. Therefore, this review aims to provide up-to-date information on miRNAs as biomarkers and cancer drugs in clinical trials.

## Cancer: Clinical trials on microRNAs’ diagnostic and therapeutic potential

The discovery of microRNAs' critical role in tumor development has sparked numerous clinical trials focused on evaluating their potential as biomarkers and targets for anti-cancer therapies. Taewan Kim from Shenzhen University, China, and Carlo Croce from The Ohio State University in Columbus, USA, summarize the various ongoing and completed trials aimed at determining whether cancer-linked microRNAs found in blood or tissues can serve as accurate predictors of cancer development or drug efficacy. The researchers discuss the development of drug mimics or inhibitors of specific microRNAs that are now in or nearing human testing as potential anti-cancer therapeutics. Although the research is still in its early stages, the authors envision a future where microRNA-based medicines and diagnostics become routine in cancer patient care, improving personalized treatment strategies and outcomes.

## Introduction

**Fig. 1:**
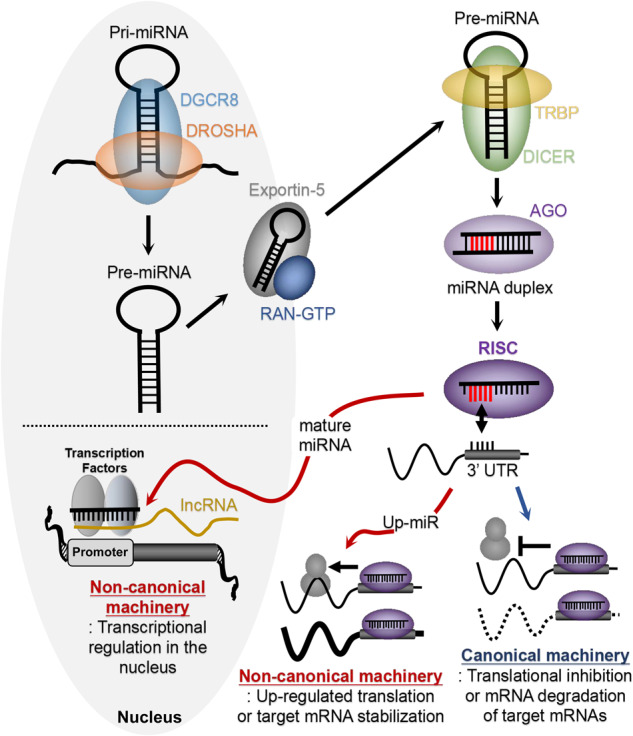
The biogenesis and the canonical and noncanonical functional machinery of microRNAs. Mature miRNAs are generated from pri-miRNAs through pre-miRNAs by implicating various proteins including DGCR8, Drosha, Exportin-5, TRBP, Dicer, and Ago. Mature miRNAs function through the canonical machinery at the posttranscriptional level or the non-canonical machinery at the transcriptional or posttranscriptional levels.

MicroRNAs (miRNAs) are one of the shortest endogenous noncoding RNAs, consisting of 20–25 nucleotides^1^. Since the discovery of the first miRNA, lin-4 in Caenorhabditis elegans (C. elegans) in 1993, miRNA processing and functional machinery have been elucidated, and thousands of miRNAs have been identified across species^1–3^. Although the expression and processing machinery of miRNAs are relatively well understood, the majority of miRNAs are processed by the canonical miRNA biogenesis pathway employing the Drosha/DGCR complex, Exportin-5/RAN-GTP complex, and Dicer/TRBP complex^4,5^. Primary miRNA transcripts (pri-miRNAs) transcribed by RNA polymerase II (Pol II) are precisely cut into precursor miRNAs (pre-miRNAs) with a stem‒loop (hairpin) structure by the Drosha/DGCR complex, and pre-miRNAs are transported into the cytosol by the Exportin-5/RAN-GTP complex. In the cytosol, the stem‒loop pre-miRNAs are edited to miRNA duplexes by the Dicer/TRBP complex, removing the loop structure. In the canonical functional mechanism, the RNA-induced silencing complex (RISC) built by Ago (Ago2 in humans), a mature miRNA strand derived from the miRNA duplex, and other accessories binds to the 3′ UTR of target mRNAs. The seed sequence, defined as the first 2–8 nucleotides of the 5′ end of miRNAs, typically contributes to the interaction between the RISC and target mRNAs (Fig. 1)^1,4,5^. Intriguingly, recent investigations have shown that some miRNAs, such as miR-212-5p and miR-221-5p, upregulate the protein levels of target mRNAs through the interaction of the canonical functional machinery with the 3′ UTR of these mRNAs, and these miRNAs are called up-miRs^6^. In addition, nuclear miRNAs employ different functional mechanisms. For instance, nuclear miR-466c activates the transcription of the VEGFA gene by interacting with long noncoding RNAs expressed from the promoter of the VEGFA gene (Fig. 1)^7^. To determine the significance of novel functional mechanisms of miRNAs, further studies are needed.

Since the discovery of miRNA, their functions have been revealed at the molecular and cellular levels. Nonetheless, there was a lack of biomedical or clinical data regarding the roles of miRNAs in human diseases. In 2002, the first evidence showing the role of miRNAs in human disease was reported. The frequently deleted 13q14.3 region in chronic lymphocytic leukemia (CLL) contains two tumor suppressor miRNAs, miR-15 and miR-16^8^. Deletion of these miRNAs in the 13q14.3 region is a critical cause of CLL development. This finding sparked active research on noncoding RNAs, including miRNAs, as causes of human diseases, including cancer. For instance, miRNAs such as mir-34 and miR-200 are upregulated by the tumor suppressor p53, which is frequently deleted, mutated, and/or inactivated in most types of cancer^9–11^. Therefore, suppression of these tumor-suppressive miRNAs by p53 inactivation promotes cancer development. On the other hand, miR-17-5p and miR-20a are transactivated by MYC, which is an oncogene that is hyperactivated and/or overexpressed in various cancer types^12^. The oncogenic miRNAs activated by MYC are involved in the initiation and progression of MYC-driven cancers. Currently, the human genome is believed to encode more than 2500 miRNAs (miRBase database, www.miRBase.org). Numerous miRNAs show unique expression patterns and context-dependent functions in different types of cancer^13^. In addition to the general functions of miRNAs in cancer, each cancer type features unique miRNA expression patterns.

The increase in research on the role of miRNAs in human diseases has allowed the identification and characterization of various miRNAs exclusively expressed or downregulated in specific human diseases^14^. As a result, a number of miRNAs have been assessed in preclinical studies and clinical trials^15^. The drug MRG-110 is an inhibitor of miR-92a^16^. Mir-92a plays an antiangiogenic role in the development of cardiovascular disease and retards wound healing in multiple organ systems^17^. Therefore, inhibition of miR-92a increases angiogenesis and improves wound repair in cardiovascular disease. In 2019, a phase 1 clinical trial of the miR-92a inhibitor MRG-110 was completed, showing the safety and efficacy of MRG-110 in humans^18^. The drug miravirsen (SPC3649) is an inhibitor of miR-122 that forms an oligomeric complex with the HCV genome to stabilize it, thus promoting HCV replication in liver cells^19^. Given the results of a preclinical study and a phase 1 clinical trial, a phase 2 clinical trial of miravirsen was completed in 2012^20^. Although more clinical trials using miravirsen have been completed or registered, the results or updates are not yet available. In addition, RGLS8429 is an inhibitor of miR-17. In autosomal dominant polycystic kidney disease (ADPKD), mir-17 accelerates kidney cyst growth by targeting mRNAs of the PKD1 or PKD2 genes^21^. Following preclinical evaluation and a phase 1 clinical trial of the first-generation miR-17 inhibitor RGLS4326, the second-generation miR-17 inhibitor RGLS8429 has been developed and tested^22,23^. The phase 1 clinical trial of RGLS8429 was completed in 2022. RGLS8429 is a very promising drug candidate for ADPKD that is currently being tested in an active clinical trial. The drug MRG-201 is also an miRNA drug that is being tested in a clinical trial; MRG-201 is a mimic of miR-29 that represses the expression of collagen and other proteins promoting scar formation^24^. To test the clinical potential of the miR-29 mimic the treatment of keloid and fibrous scar formation, a phase 1 clinical trial was completed in 2021. In addition, MRG-229, an updated miR-29 mimic with improved chemical stability, was developed and tested as a treatment for pulmonary fibrosis in the preclinical stage^25^. Mimics of miR-466c increase VEGFA expression, and they are also being tested in the preclinical stage as a treatment for peripheral artery disease (PAD) and heart failure (HF)^7^.

Similar to the examples of miRNA therapies in other diseases, various mimics or inhibitors of miRNAs have been clinically tested in cancer^24^. Though a number of preclinical and clinical trials were in progress, some of them were interrupted because of clinical and business issues. Nevertheless, several studies of clinical applications of miRNA therapy are ongoing. In this review, we introduce miRNA therapies currently in the preclinical or clinical stage of development for cancer treatment and diagnosis.

## Mirnas as biomarkers of cancer diagnosis and drug efficacy prediction

Many clinical trials examining the clinical potential of miRNAs as biomarkers for cancer therapy response prediction, diagnosis, and prognostication are underway. As diagnostic markers, miRNAs in blood or tissue samples from cancer patients have been tested. A few clinical trials of diagnostic miRNAs were recently completed, although the results have not been reported. For instance, miR-155 was used to diagnose non-muscle-invasive bladder cancer (ClinicalTrials.gov identifier: NCT03591367), and miRNA profiling was used to predict the development of multicentric breast cancer (ClinicalTrials.gov identifier: NCT04516330). A number of additional diagnostic clinical trials using patient blood or tissue samples are ongoing. The ability of let-7a and miR-124 to diagnose non-Hodgkin’s lymphoma and acute leukemia is being tested (ClinicalTrials.gov identifier: NCT05477667). Plasma miRNAs are also being profiled to discover novel diagnostic miRNAs in lung and gynecologic cancers (ClinicalTrials.gov identifier: NCT02247453 & NCT03776630). In addition, the ability of some miRNAs to characterize unclassified cancer cell types is being tested in clinical trials. The ability of ten miRNAs to define and characterize undetermined types of thyroid cancer is being tested (ClinicalTrials.gov identifier: NCT04285476). Interestingly, novel miRNA markers for colorectal cancer screening are being searched for in fecal samples (ClinicalTrials.gov identifier: NCT05346757). Clinical trials is to study miRNA markers regulated by probiotics are also underway. Probiotic supplementation with Helicobacter pylori (H. pylori) eradication promotes the regression of intestinal metaplasia by regulating the Wnt/beta-catenin signaling pathway^26–31^. Because miRNAs play crucial roles in regulating the Wnt/beta-catenin signaling pathway in gastric carcinogenesis, probiotics may modulate miRNAs implicated in the Wnt/beta-catenin signaling pathway and gastric carcinogenesis^32–50^. Therefore, one clinical trial (ClinicalTrials.gov identifier: NCT05544396) aimed to identify and characterize miRNA markers regulated by probiotics in gastric carcinogenesis. In addition to these clinical trials, more clinical trials aiming to identify miRNAs or test the diagnostic utility of miRNA biomarkers in cancer have been completed or are ongoing according to ClinicalTrials.gov.

The ability of miRNAs to predict the efficacy of various cancer therapies has also been studied. MiRNAs are being clinically profiled as predictive markers of chemotherapeutic efficacy in metastatic castration-resistant prostate cancer (ClinicalTrials.gov identifier: NCT04662996). To improve the efficacy of adjuvant therapy in colon cancer, six miRNAs (miR-21, miR-20a, miR-103a-3p, miR-106b, miR-143, and miR-215) are under clinical investigation (ClinicalTrials.gov identifier: NCT02466113). The potential of miRNA markers to predict targeted immunotherapy efficacy has also been examined. In NSCLC, exosomal miRNAs were profiled and characterized before or after administration of immunotherapy targeting PD-1 or PD-L1 (ClinicalTrials.gov identifier: NCT04427475). To examine the ability of miRNAs to predict the efficacy of multiple chemotherapy drugs (epirubicin, cyclophosphamide, paclitaxel, and carboplatin), the correlation between blood miRNA levels and drug resistance was studied in triple-negative breast cancer (ClinicalTrials.gov identifier: NCT04771871). MiR-371a-3p in serum is being evaluated as a marker of resistance to chemical drugs (carboplatin, etoposide, and cisplatin) and radiotherapy in testicular germ cell tumors (ClinicalTrials.gov identifier: NCT05529251). The correlation of miR-141 and miR-375 with radiation resistance is also being investigated in prostate cancer (ClinicalTrials.gov identifier: NCT02391051). Because many lines of evidence have shown that many miRNAs are involved in resistance to various therapeutic modalities and strategies in cancer, more clinical trials evaluating the utility of miRNAs as predictive and prognostic markers of cancer treatments should be developed^51–57^.

Since the discovery of miRNA functions in cancer, many miRNAs have been identified in various cancer types^8,13^. Furthermore, additional miRNAs have been found to be specifically dysregulated in different cancer types because of the discovery of novel miRNAs and the development of new detection technologies over the last decade^58–61^. For example, miR-34697, miR-45165, miR-638, and miR-152 have been identified to play a role in non-small cell lung cancer (NSCLC); miR-885-5p has been identified to play a role in kidney cancer; and miR-1285 has been identified to play a role in prostate cancer^58,62^. More recently, it has been revealed that genetic and epigenetic modifications of miRNAs can be used as diagnostic and prognostic markers in cancer. The single nucleotide polymorphisms found in miRNAs are associated with cancer susceptibility^63^. ADAR proteins frequently participate in A-to-I editing, making them important diagnostic and prognostic markers because they modulate miRNA processing, expression, and activity in cancer^64,65^. In addition to A-to-I editing, N6-methyladenosine (m6A) modification of miRNAs is a critical epigenetic modification associated with cancer diagnosis and prognosis. The development of new technologies such as MeRIP-Seq (m6A-seq) has led to the identification of miRNAs with m6A modifications^66^. MiRNA methylation is also associated with prognosis and drug resistance in cancer^67,68^. Therefore, in addition to the miRNAs identified in early miRNA research, newly discovered miRNAs and miRNA modifications are being recognized as potential diagnostic and prognostic markers for clinical trials.

## Mirnas as cancer therapeutic targets

### MiRNA-34a

The miR-34 family members, including miR-34a, miR-34b, and miR-34c, were highlighted as p53-regulated tumor suppressor miRNAs in 2007^9,10^. The primary transcripts of miR-34 family members are directly transactivated by the transcription factor p53, and members of the miR-34 family that are induced by the tumor suppressor p53 modulate the effects of p53 on the cell cycle, cell growth, apoptosis, and the DNA damage response^69^. The tumor suppressor p53 is frequently mutated, deleted, and/or downregulated in most cancer types. miR-34a is upregulated by p53 and is a main member of the miR-34 family; miR-34a is correspondingly downregulated in various cancers^70^. For these reasons, miR-34a is one of the most promising candidates for miRNA drugs in cancer.

The drug MRX34 is a synthetic double-stranded miR-34a mimic encapsulated in a liposomal nanoparticle. The phase 1 clinical trial (ClinicalTrials.gov identifier: NCT01829971) of MRX34 was the first-in-human clinical trial of miRNA therapy^71^. In the clinical trial, the drug was tested in various cancer types, such as primary liver cancer, small-cell lung cancer (SCLC), lymphoma, melanoma, multiple myeloma, renal cell carcinoma, and NSCLC. However, the clinical trial was terminated owing to serious immune-mediated adverse events resulting in four patient deaths^72,73^. As a consequence, another clinical trial (ClinicalTrials.gov identifier: NCT02862145) using MRX34 combined with dexamethasone in melanoma was withdrawn. The first trials of miRNA therapy provided critical lessons, although the clinical trial using MRX34 did not achieve dramatic success. Exogenous miRNA mimics require further development and improvement to avoid toxicity in humans. In the trial, the liposomal delivery system was not the cause of the immune-related adverse events^73^. Furthermore, a tumor-targeted delivery system could reduce the off-target toxicity of miRNA drugs. Despite the side effects, treatment with MRX34 decreased the expression of miR-34 target genes, oncogenes, and immune escape-related genes in cancer patients^72,73^. Therefore, if improvements in synthetic miRNA mimics and delivery systems can be made, miR-34a is still a promising target of miRNA cancer therapy. Consequently, miRNA therapy has potential as a next-generation therapy despite the early termination of the MRX34 clinical trial.

### MiRNA-16

MiR-16 and miR-15 were the first two miRNAs revealed to cause human diseases, in particular, cancer^8^. Mir-16 (miR-16-1) and miR-15 (miR-15a) are clustered at chromosome 13q14.3, which is frequently deleted in various types of cancer^74^. Monoallelic or biallelic deletion of the 13q14.3 region is the most common cytogenic abnormality in CLL and is found in more than 50% of CLL cases^8^. In addition, deletion of 13q14.3 is frequently found in other cancer types: ~50% of mantle cell lymphoma, ~60% of prostate cancer, and 16–20% of multiple myeloma cases^8,75^. Although several genes, including DLEU1, DLEU2, TRIM13, KCNRG, and SPRYD7, are localized at the 13q14.3 region, those genes do not show consistent tumor suppressor functions^8^. The discovery of the presence of the miR-16-1/15a cluster in the intron of the DLEU2 gene revealed a potential mechanism by which 13q14.3 deletion is associated with cancer development^8^. In particular, miR-16 and miR-15 suppress the translation and stability of BCL2 mRNA, inhibiting cell apoptosis^76^. Consequently, deletion of the 13q14.3 region, which includes the tumor suppressors miR-16 and miR-15, increases the expression of the oncogene BCL2^74^. In addition to BCL2, a number of genes, including ROR1, RPS6KB1, WIP1 (PPMID), MCL-1, CHK1, WEE1, CCND1, CCND2, CCNE1, E2F, WNT3A, STAT-3, VEGF, and BMI-1, are known as targets of miR-16 and miR-15^77^. Tumor-suppressor roles of miR-16 and miR-15 were also revealed in other cancers, including lung cancer, malignant pleural mesothelioma (MPM), nasopharyngeal cancer, breast cancer, squamous cell adenocarcinoma, retinoblastoma, and gastric cancer. Although 13q14.3 deletion is not as frequent in these cancer types as it is in CLL, miR-16 is also frequently downregulated in these cancer types^77^. Another miR-16/15 cluster is also found in chromosome 3 in the intron of the SMC4 gene. This miR-16/15 cluster at chromosome 3 may play a critical role with the cluster in chromosome 13q^77^.

The drug TargomiR is a synthetic double-stranded mimic of miR-16 encapsulated by a bacterial minicell system known as EnGeneIC Dream Vectors (EDVs)^78^. In particular, TargomiR employs the EGFR-targeting EDV system to precisely deliver miR-16 mimics into EGFR-overexpressing tumor cells in patients with recurrent MPM or NSCLC. In the second-in-human clinical trial (ClinicalTrials.gov identifier: NCT02369198) of miRNA therapy, the improved synthetic double-stranded RNA mimic and delivery system were better tolerated and showed early signs of tumor suppression^78^. Although the result was not dramatic and there were minor adverse events in this clinical trial, the results provide clues for successful miRNA therapy. In the MRX34 clinical trial, the double-stranded miRNA mimics and the dose were thought to be major causes of immune-related toxicity^72,73^. However, the relative success of the clinical trial of the double-stranded mimic TargomiR suggests that the carrier system is more responsible for the inflammatory toxicities^35^. In addition, the moderate response seen in the clinical trial might be due to the low dose of miR-16 mimics. Overall, the results of this trial indicate that a more cancer-specific and safe delivery system is needed for miRNA therapy.

### MiRNA-155

Mir-155 is an oncogenic miRNA overexpressed in lymphoma, leukemia, and most solid cancers^79,80^. The expression of miR-155 is also associated with poor prognosis in various cancers. The functions of miR-155 have been reported not only in cancer but also in other diseases, including viral infection, immune diseases, neurological diseases, diabetes, and cardiovascular diseases^81^. Mir-155 is processed from the primary transcript miR-155HG (host gene), which was formerly known as BIC (B-cell integration cluster)^80^. Like many other miRNAs, miR-155 is conserved between humans and mice^79^. Therefore, the functions of miR-155 have been derived from mouse models as well as in vitro cell models^81^. Studies using in vivo models of miR-155 have shown that the critical roles of miR-155 in inflammatory diseases are directed by impairment of immune cells, including B cells, T cells, mast cells, dendritic cells, and macrophages. In particular, transgenic mouse models of miR-155 show uncontrolled T-cell proliferation, abnormal natural killer cell development, and myeloproliferative disorders, validating the oncogenic role of miR-155^81,82^. Indeed, administration of miR-155 inhibitor led to the depletion of tumorigenic lymphoid cells in vivo, suggesting that miR-155 is a promising target miRNA for treating leukemia and lymphoma^83^.

The drug MRG-106 (cobomarsen) is an miR-155 inhibitor synthesized as a locked nucleic acid (LNA)-modified oligonucleotide^84^. A phase 1 clinical trial (ClinicalTrials.gov identifier: NCT02580552) tested MRG-106 in cutaneous T-cell lymphoma (CTCL), CLL, diffuse large B-cell lymphoma (DLBCL), and adult T-cell leukemia/lymphoma (ATLL)^83^. Given the successful results of the phase 1 clinical trial, a phase 2 clinical trial (ClinicalTrials.gov identifier: NCT03713320) in CTCL was developed. Unfortunately, the phase 2 clinical trial was terminated early due to business reasons of the sponsored company according to the updated information on ClinicalTrials.gov. This clinical trial was the first trial using miRNA inhibitors, unlike the previous two clinical trials using miRNA mimics. A single miRNA can have broad functions by targeting numerous targets. Therefore, as shown in the previous two clinical trials of the tumor suppressors miR34 and miR-16, the mimics are likely to cause unintended side effects if the strategy lacks mechanisms for specific delivery and targeting of cancer cells. On the other hand, the targeting of oncogenic miRNAs using miRNA inhibitors may at least partly avoid the risk of exogenous overexpression of specific miRNAs as long as the inhibitors are specific to the target miRNAs. Because the early termination of the phase 2 clinical trial for MRG-106 was not caused by issues of safety or efficacy, this drug may still be tested in further trials and may be promising for clinical applications in the future.

### MiRNA-193a-3p

MiR-193 consists of miR-193-3p and miR-193-5p, which are derived from the 3′ arm and the 5′ arm, respectively, of the stem‒loop structure of premiR-193 (most miRNAs in this review are derived from the 5′ arm unless otherwise indicated)^85^. PremiR-193 is expressed from two loci: miR-193a on chromosome 17 and miR-193b on chromosome 16^85^. The sequences of miR193 family members, including miR-193a-5p, miR-193a-3p, miR-193b-5p, and miR-193-3p, are homologous but slightly different, leading to differential mRNA targeting^85^. Members of the miR-193 family were studied as potential modulators of the apoptosis pathway^86^. Indeed, overexpression of miR-193 family members induces apoptosis by indirectly activating caspase-3 and caspase-7^87^. However, it was also reported that miR-193 accelerated the proliferation of mesenchymal stem cells^88^. This could be due to the unique sequence- and cell context-dependent roles of the miR-193 family members. Nonetheless, most lines of evidence indicate a tumor-suppressive role of miR-193 family members, in particular, miR-193a-3p, in a variety of cancers, including breast cancer, lung cancer, colorectal cancer, squamous cell carcinoma, melanoma, acute leukemia, osteocarcinoma, pleural mesothelioma, and thyroid carcinoma^85^. Although the function of miR-193a-3p has not been specifically defined, miR-193 also represses the growth of gastric cancer, endometrial carcinoma, hepatocellular carcinoma (HCC), ovarian cancer, pancreatic cancer, and prostate cancer tumors^85^. Consistently, miR-193a-3p is often downregulated in the tissues of diverse cancers compared to their adjacent normal tissues^85^.

The drug INT-1B3 is a lipid nanoparticle (LNP)-formulated miR-193a-3p mimic (1B3)^89^. A phase 1 clinical trial (ClinicalTrials.gov identifier: NCT04675996) of INT-1B3 is ongoing. The function of the novel synthetic miR-193a-3p mimic 1B3 was tested in cell lines derived from several cancers, such as triple-negative breast cancer (TNBC), NSCLC, melanoma, colon cancer, and HCC. Treatment with 1B3 resulted in the upregulation of the tumor-suppressive PTEN pathway and the downregulation of many oncogenic pathways in cancer-derived cells^90^. In addition, despite the different genetic backgrounds of these cancer cell lines, 1B3 showed consistent effects in suppressing cell proliferation, the cell cycle, and cell migration and inducing apoptosis, cell senescence, and DNA damage^91^. These results suggest the potential of IB3 in a broad range of cancers. Given the notable effects of 1B3, a novel LNP formulation of 1B3 was developed (INT-1B3). Studies of INT-1B3 in orthotopic mouse models have revealed that INT-1B3 can be safely and efficiently delivered to tumors in vivo^89^. Following these successful preclinical studies, INT-1B3 is currently being tested in a phase 1 clinical trial to determine the maximally tolerated dose, safety, pharmacokinetics, pharmacodynamic response, and antitumor activity in patients with various solid cancers. In addition to INT-1B3, another LNP-formulated tumor-suppressive miRNA mimic, INT-5A2, is under development for HCC and glioblastoma therapy (https://interna-technologies.com). The identity of the miRNA that INT-5A2 mimics has not yet been publicly released.

### MiRNA-10b

MiR-10b is a member of the miR-10 family. The miR-10 family resides in the evolutionarily well-conserved HOX gene cluster^91^. Similar to the HOX gene, miR-10 family members are also highly conserved across species. MiR-10a is localized in the intron of the HOXB3 gene in chromosome 17, and miR-10b is found near the HOXD4 gene in the HOXD cluster in chromosome 2. In cancer research, miR-10b has drawn considerable attention as a key regulator of tumor invasion and metastasis in breast cancer. Mir-10b, which is transcriptionally activated by epithelial-mesenchymal transition (EMT) induced by the transcription factor TWIST, indirectly increases the expression of the prometastatic gene RHOC by directly targeting and suppressing HOXD10 mRNAs^92^. In addition to the HOXD10 gene, various genes, including HOXB1, HOXB3, NF1, KLF4, and TIAM1, have been revealed as miR-10b targets regulating the invasion, migration, and metastasis of cancer cells^91^. In several primary cancers, such as glioblastoma, pancreatic cancer, and esophageal cancer, miR-10b is upregulated, supporting the oncogenic role of miR-10b. On the other hand, in primary breast cancer, miR-10b is not upregulated. Interestingly, however, miR-10b is highly upregulated in metastatic breast cancers, indicating the critical role of miR-10b in cancer metastasis. The upregulated expression of miR-10b in nasopharyngeal metastatic carcinoma cells and neurofibromatosis type 1 metastatic cells also supports the critical role of miR-10b in cancer metastasis^91^. Consistent with these findings, an miR-10b inhibitor (antagomir) efficiently prevented metastasis of breast cancer in a mouse model^93^.

The drug TTX-MC138 is an miR-10b inhibitor conjugated with advanced dextran-coated iron oxide nanoparticles (https://www.transcodetherapeutics.com). Recent findings showed safe and noticeable effects of LNA-based miR-10b inhibitors (antagomirs) conjugated to magnetic nanoparticles in vitro and in vivo, with the inhibitors significantly decreasing the high expression of miR-10b in breast cancer. The miR-10b inhibitor TTX-MC138 was developed based on these promising results and advances in nanotechnology. TTX-MC138 is currently being tested in the preclinical stage for the treatment of metastatic breast cancer^94–96^. MiR-10b is frequently overexpressed and plays an oncogenic role not only in metastatic breast cancer but also in other cancers^91,97^. Not surprisingly, a clinical trial (ClinicalTrials.gov identifier: NCT01849952) to assess miR-10b expression in patients with several subtypes of brain cancer is also ongoing. Prior to the development of the drug TTX-MC138, another miR-10b inhibitor, RGLS5579, was developed and preclinically tested for glioblastoma multiforme (GBM)^98,99^. Although clinical trials of RGLS5579 have not yet been initiated, RGLS5579 combined with temozolomide (TMZ) safely and meaningfully extended the survival of an orthotopic mouse model of GBM^100^. Based on the outcome of RGLS5579 treatment, TTX-MC138 also has the potential to treat various primary cancers, including brain cancer, in addition to metastatic breast cancer. Indeed, TTX-MC138 is being preclinically tested in GBM, pancreatic cancer, SCLC, and osteosarcoma according to the information provided by the company working on TTX-MC138 (https://www.transcodetherapeutics.com).

## Conclusion and perspectives

During the past two decades and since the discovery of miRNAs as a direct cause of human diseases, numerous miRNAs have been identified and characterized as oncogenic or tumor-suppressive miRNAs in almost all types of cancer and many other human diseases^4,8,13,14,31^. As a result, a large number of preclinical and clinical trials centered on miRNAs have been carried out during the last decade, as cataloged by ClinicalTrials.gov. Studies profiling miRNAs have identified cancer-specific miRNAs in most types of cancer, describing the potential of miRNAs as diagnostic markers^13,31^. Bioinformatic and biostatistical analyses have also revealed a strong association of cancer-specific miRNAs with clinical outcomes, including prognosis, survival, and drug resistance^101–104^. Hence, clinical trials of the utility of miRNAs in diagnosis have been initiated. MiRNAs are abundant noncoding RNAs, and their short length increases their stability compared with longer RNA molecules, which are readily broken down by ribonuclease^105–107^. Furthermore, miRNAs are secreted into extracellular fluid alone or encapsulated by vesicles such as microvesicles and exosomes^100,107^. Subsequently, the secreted miRNAs are found in the blood circulation^108^. In summary, cancer-specific and circulating miRNAs are attractive diagnostic markers. In addition to diagnostic miRNAs, miRNAs that can be used to predict drug efficacy and patient prognosis will significantly aid the advancement of precision cancer medicine. Correspondingly, as introduced in this review, many clinical trials of diagnostic and predictive miRNAs are ongoing in various cancer types.

Several clinical trials of improved miRNA drug strategies, such as synthetic RNA molecules and advanced delivery technologies, are ongoing despite the failure of the first-in-human clinical trial of miRNA cancer therapy (Table 1)^15^. Due to the pleiotropic function of miRNAs, targeting miRNAs may be challenging and cause unexpected side effects, as shown in the first trial with tumor-suppressive miR-34a mimics^15,24,109^. However, cancer is not a single disease caused by a single genetic mutation or epigenetic alteration. Cancer cells develop via the accumulation of numerous genetic mutations and epigenetic alterations at the genome level^110^. Indeed, as confirmed by the limited success of many cancer therapeutic strategies aiming at a single or few targets, cancer is unlikely to be cured by targeting one or a few factors^95,110^. Thus, the unique pleiotropic effects of miRNAs make them an attractive therapy type. Treatment with a single miRNA drug can have a similar effect as treatment with multiple drugs^24^. There remain challenges with miRNA therapy such as toxicity, adverse effects, and low efficacy at a high dose that need to be resolved^15,24^. However, the advancement of biochemistry and bioengineering technologies has made it possible to address the obstacles in miRNA therapy as described in this review. Recent preclinical and clinical trials have shown higher safety, efficacy, and specificity of new synthetic RNA oligomers for miRNA mimics and antisense miRNA inhibitors^35,41,46^. In addition, new promising delivery systems have been developed and applied for the effective and precise delivery of miRNA drugs^35,46,51–53^. As expected, miRNA-focused preclinical and clinical trials are common in multiple human diseases, including cardiovascular diseases. Given the relevant progress of miRNA therapy, miRNA therapy will likely be a leading next-generation cancer therapy with the application of advanced synthetic RNA technologies and cancer-specific delivery systems.

**Table 1:** Summary of miRNA drugs for cancer in preclinical or clinical trials.

Drug	MiRNA	Drug type	Delivery & combination	Phase	ClinicalTrials.gov Identifier	Cancer type
MRX34	miR-34a	Double-stranded mimic	Liposome nanoparticle	Phase 1 (Terminated)	NCT01829971	Liver cancer, SCLC, lymphoma, melanoma, renal cell carcinoma, NSCLC
			Liposome nanoparticle + Dexamethasone	Phase 1 (Withdrawn)	NCT02862145	Melanoma
TargomiR	miR-16	Double-stranded synthetic mimic	Bacterial minicell (EDVs)	Phase 1 (Completed)	NCT02369198	MPM, NSCLC
MRG-106 (Cobomarsen)	miR-155	LNA-based inhibitor	N/A	Phase 1 (Completed)	NCT02580552	CTCL, CLL, DLBCL, ATLL
				Phase 2 (Early termination)	NCT03713320	CTCL
INT-1B3	miR-193a-3p	Mimic (1B3)	Lipid nanoparticle (LNP)	Phase 1 (Recruiting)	NCT04675996	TNBC, NSCLC, melanoma, colon cancer, HCC
TTX-MC138	miR-10b	Inhibitor (Antisense oligo)	Dextran-coated iron oxide nanoparticles	Preclinical	N/A	Metastatic breast cancer, GBM, pancreatic cancer, SCLC, osteosarcoma
RGLS5579	miR-10b	Inhibitor (Antisense oligo)	Unknown	Preclinical	N/A	GBM
